# Fitness Cost of Aflatoxin Production in Aspergillus flavus When Competing with Soil Microbes Could Maintain Balancing Selection

**DOI:** 10.1128/mBio.02782-18

**Published:** 2019-02-19

**Authors:** Milton T. Drott, Tracy Debenport, Steven A. Higgins, Daniel H. Buckley, Michael G. Milgroom

**Affiliations:** a School of Integrative Plant Science, Plant Pathology and Plant-Microbe Biology Section, Cornell University, Ithaca, New York, USA; b School of Integrative Plant Science, Soil and Crop Sciences Section, Cornell University, Ithaca, New York, USA; University of California, Irvine

**Keywords:** aflatoxin, secondary metabolism, *Aspergillus flavus*, fitness cost

## Abstract

Selective forces that maintain the polymorphism for aflatoxigenic and nonaflatoxigenic individuals of Aspergillus flavus are largely unknown. As soils are widely considered the natural habitat of A. flavus, we hypothesized that aflatoxin production would confer a fitness advantage in the soil environment. To test this hypothesis, we used A. flavus DNA quantified by quantitative PCR (qPCR) as a proxy for fitness of aflatoxigenic and nonaflatoxigenic field isolates grown in soil microcosms. Contrary to predictions, aflatoxigenic isolates had significantly lower fitness than did nonaflatoxigenic isolates in natural soils across three temperatures (25, 37, and 42°C). The addition of aflatoxin to soils (500 ng/g) had no effect on the growth of A. flavus. Amplicon sequencing showed that neither the aflatoxin-producing ability of the fungus nor the addition of aflatoxin had a significant effect on the composition of fungal or bacterial communities in soil. We argue that the fitness disadvantage of aflatoxigenic isolates is most likely explained by the metabolic cost of producing aflatoxin. Coupled with a previous report of a selective advantage of aflatoxin production in the presence of some insects, our findings give an ecological explanation for balancing selection resulting in persistent polymorphisms in aflatoxin production.

## Abstract (precis)

Aflatoxin, produced by the fungus Aspergillus flavus, is an extremely potent hepatotoxin that causes acute toxicosis and cancer, and it incurs hundreds of millions of dollars annually in agricultural losses. Despite the importance of this toxin to humans, it has remained unclear what the fungus gains by producing aflatoxin. In fact, not all strains of A. flavus produce aflatoxin. Previous work has shown an advantage to producing aflatoxin in the presence of some insects. Our current work demonstrates the first evidence of a disadvantage to A. flavus in producing aflatoxin when competing with soil microbes. Together, these opposing evolutionary forces could explain the persistence of both aflatoxigenic and nonaflatoxigenic strains through evolutionary time.

## INTRODUCTION

Although there is a large body of research focused on the role of microbial secondary metabolites *in vitro*, the ecological functions of these compounds remain poorly understood. Even the ecological role of antibiotics is being questioned because soils, the natural environment for many of the microbes that produce antibiotics, are now commonly thought to contain subinhibitory concentrations of these compounds (1, 2). Beyond antibiotics, few microbial secondary metabolites have received as much attention as aflatoxin, which is produced by the fungi Aspergillus flavus, Aspergillus parasiticus, and a few other closely related Aspergillus species. Aflatoxin is an extremely potent hepatotoxin that causes acute toxicosis, cancer, immune suppression, and stunted growth in children (2–6). Aflatoxin contamination of corn, peanuts, cotton, tree nuts, and other crops is estimated to cost hundreds of millions of dollars annually in the United States alone (7). However, not all strains of A. flavus produce aflatoxin. Extensive field sampling in the United States found that 29% of all A. flavus isolates did not produce aflatoxin (8). Worldwide, the two chemotypes (fungal isolates that produce and do not produce aflatoxin, known as aflatoxigenic and nonaflatoxigenic, respectively) are often found in soil in the same field (9). Nucleotide sequence analysis of 21 regions in the aflatoxin gene cluster in A. flavus and A. parasiticus confirmed that the polymorphism for aflatoxin production is maintained by balancing selection (10, 11). As with antibiotics, however, attempts to understand the forces selecting for (and against) aflatoxin production have been done using *in vitro* laboratory assays that are not representative of conditions the fungus would encounter in its natural habitat.

To maintain the polymorphism for aflatoxin production by balancing selection, aflatoxigenic individuals must be favored under some conditions while nonaflatoxigenic individuals are favored under others. Without selection favoring each chemotype under different conditions, one of the chemotypes should become fixed and the polymorphism should not be maintained. Janzen (12) hypothesized that aflatoxin production is favored in the presence of insects, birds, mammals, or soil microbes through interference competition. Under this hypothesis, the toxic effects of aflatoxin produced in nutrient-rich substrates like seeds increases the fitness of fungi producing them by deterring competitors. Recently, Drott et al. (13) provided evidence that aflatoxin production increased the fitness of A. flavus in the presence of some insects but not in their absence. They speculated that A. flavus might incur a fitness cost associated with the biosynthesis of aflatoxin in the absence of insects because it is energetically costly to produce (14–16). However, if there were a cost, it may have been masked in these experiments (13) because a nutrient-rich medium was used or because the differences may have been too small to detect experimentally. Nonetheless, the fitness advantage observed in the presence of insects, together with the proposed cost of production, could drive balancing selection as a function of the presence of insects and their susceptibility to aflatoxin. This explanation for the balancing selection acting on aflatoxin production under these conditions does not, however, preclude the toxin from having benefits (or costs) in other environments.

Soil is widely considered the natural habitat of A. flavus and is thus a likely environment for aflatoxin production to benefit the fungus through interference competition with other microbes. However, little is known about the ecology of the fungus or the role of aflatoxin in soil environments (9, 17–19). A. flavus actively colonizes organic matter in or on soil with little growth through the soil itself (20). Both aflatoxigenic and nonaflatoxigenic chemotypes of A. flavus are commonly isolated from agricultural soils (8, 9). It had been thought that lower latitudes favor aflatoxigenic strains (21). Indeed, Wicklow et al. (20) speculated that the higher frequencies of aflatoxigenic isolates in soil at these latitudes may be correlated with greater densities of soil insects associated with a relatively warm climate. However, Drott et al. (22), sampling A. flavus from two north-south transects in the United States, found that while population densities of A. flavus increase at lower latitudes, there was no clear pattern to the frequency of aflatoxigenic isolates across latitudes. Alternatively, if aflatoxin production were favored at higher temperatures, high rates of migration observed in the United States potentially could erase geographic patterns of selection. To clarify this lack of geographic population structuring, we hypothesize that interference competition from microbial communities may select for aflatoxigenic individuals in warmer soils. Under the interference competition hypothesis, aflatoxigenic isolates of A. flavus would suppress some microbes, thus making the composition of the soil microbial community more favorable for its own growth. Consistent with this hypothesis, there appears to be a relationship between aflatoxin production and soil microbes, as aflatoxin production is induced by several soil bacteria and yeasts *in vitro* (23, 24). Moreover, expression of aflatoxin biosynthetic genes in A. flavus and aflatoxin *per se* have been observed in soil (25). Little is known, however, about the impact of aflatoxin production on the fitness of A. flavus and the composition of the microbial community in soil.

Support for the hypothesis that aflatoxin mediates interference competition with soil microbes from laboratory studies is mixed. Aflatoxin has been reported as having limited impact on soil microbes *in vitro*, having little to no effect on growth even at concentrations well above those observed in contaminated agricultural commodities (26, 27). In contrast, however, Angle and Wagner (28) showed that at high concentrations (10,000 ppb), aflatoxin B_1_ reduced the number of viable fungal and bacterial propagules *in vitro*. Such decreases in microbial population density could be interpreted as support for the idea of interference competition being mediated by aflatoxin. However, that study did not assess the fitness of A. flavus or identify specific ecologically relevant microbial species. Though methods are now available to estimate fitness and assess the composition of microbial communities in soil, we still lack information on specific microbes interacting with A. flavus or being inhibited by aflatoxin. Several studies have demonstrated the mutual inhibition of A. flavus and various Bacillus species (23, 27, 29). Some of these same Bacillus species are more sensitive to the antibiotic effects of aflatoxin than most other bacteria (23, 27, 29). Interestingly, both A. flavus and Bacillus populations are largest in field soils with persistent drought and high-temperature stress environments (>35°C) (9), potentially providing an environment for direct competition between these microbes. These studies, done either in the absence of the fungus and/or *in vitro*, leave open the possibility that aflatoxin acts through interference competition to confer a fitness advantage when competing with soil microbes, much as it does in the presence of some insects (13).

Our main objective was to test the hypothesis that interference competition with soil microorganisms by the production of aflatoxin confers a fitness advantage to A. flavus and thus partially explains balancing selection for aflatoxin production. Specifically, we addressed the following two questions: (i) does the production or addition of aflatoxin increase the fitness of A. flavus in soil? and (ii) does the production of aflatoxin by A. flavus or the addition of aflatoxin in soil affect microbial community composition? We used culture-independent methods to determine the effect of aflatoxin on both the fitness of A. flavus during its interaction with soil microbes in field-soil microcosms and the composition of microbial communities. We compared naturally occurring aflatoxigenic and nonaflatoxigenic strains of A. flavus in sterile and nonsterile (natural) soil to examine the effects of soil microbes on the fitness of A. flavus.

## RESULTS

### Effects of aflatoxin on fitness of A. flavus in sterile and natural soils. (i) Experiment 1.

**FIG 1:**
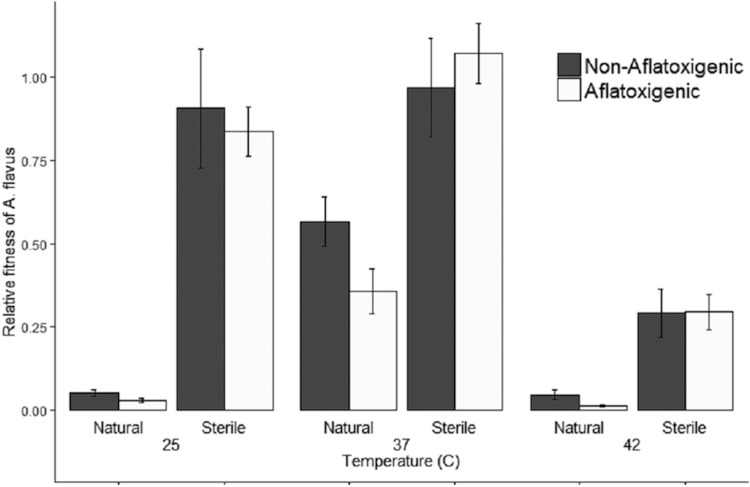
Mean relative fitness of aflatoxigenic (*n *=* *4) and nonaflatoxigenic (*n *=* *3) isolates of *Aspergillus flavus* in natural and sterile field soils at three temperatures (experiment 1). The fitness of *A. flavus* was estimated by qPCR to quantify DNA relative to a standard curve. Each treatment was replicated twice and incubated for 4 days. Error bars represent the standard error (SE). Aflatoxigenic isolates had lower fitness than nonaflatoxigenic isolates in natural soils but not in sterile soils, as indicated by the significant interaction between toxin-producing ability and soil sterility (*P = *0.043).

In experiment 1, we observed a significant interaction of soil sterility and temperature with respect to their effects on the fitness of A. flavus (analysis of variance [ANOVA], F_2,67_ = 12.8, *P < *0.0001; see Table S1 in the supplemental material). This interaction is evident in the difference in average fitness between sterile and natural soils, which is smaller at 37°C and 42°C (regardless of chemotype) and larger at 25°C (Fig. 1). Although we did not observe any difference between aflatoxigenic and nonaflatoxigenic isolates at any specific temperature (Tukey *post hoc*, *P > *0.616), there was a significant interaction between chemotype and soil sterility independent of temperature (ANOVA, F_1,67_ = 4.2, *P = *0.043; Table S1). This interaction manifests with aflatoxigenic isolates having lower fitness than nonaflatoxigenic isolates in natural soils, but not in sterile soils, across all temperatures (Fig. 1). While there was no difference in fungal fitness between sterile soils incubated at 25 and 37°C (Tukey *post hoc*, *P > *0.928), fitness was reduced 93% in natural soils incubated at 25°C compared to fitness in natural soils at 37°C (Tukey *post hoc*, *P < *0.0001) for both chemotypes.

10.1128/mBio.02782-18.3 TABLE S1 Results of ANOVA mixed linear models of fungal fitness (quantifying fungal DNA as a proxy for fitness) as determined using temperature (temp), sterility (sterility), aflatoxin-producing ability of the fungus (chemotype), added aflatoxin (PPB), experiment (experiment) and population that isolates are from (population) as determined by Drott et al. (22) and the random effect of fungal isolate. Download Table S1, DOCX file, 0.02 MB. Copyright © 2019 Drott et al. 2019 Drott et al. This content is distributed under the terms of the Creative Commons Attribution 4.0 International license.

### (ii) Experiment 2.

**FIG 2:**
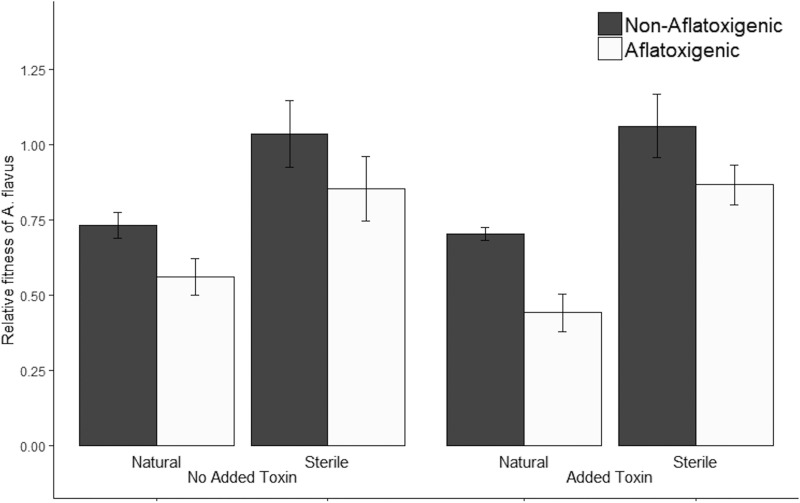
Mean relative fitness of aflatoxigenic (*n *=* *7) and nonaflatoxigenic (*n *=* *4) isolates of *Aspergillus flavus* in natural and sterile field soils with and without 500 ng/g soil (ppb) aflatoxin added (experiment 2). Fitness of *A. flavus* was estimated by qPCR relative to a standard curve. Each treatment was replicated twice and incubated for 4 days. Error bars represent the SE. The fitness of aflatoxigenic isolates was significantly lower than that of nonaflatoxigenic isolates in natural soils (*P = *0.049) but not in sterile soils (*P = *0.271), with no effect of added aflatoxin in either (*P > *0.124).

Because the fitness of A. flavus in natural soils was observed to be greatest at 37°C, we performed a second experiment in which we further investigated the effect of aflatoxin production and aflatoxin added to the soil (500 ng/g soil [ppb]) on A. flavus fitness at this temperature. Again, we observed that aflatoxigenic isolates had significantly lower fitness than nonaflatoxigenic isolates at 37°C in natural soils (ANOVA, F_1,9_ = 5.2, *P = *0.049; Table S1) but not in sterile soils (ANOVA, F_1,9_ = 1.4, *P = *0.271; Table S1; Fig. 2). This fitness cost was not influenced by the addition of aflatoxin to either natural (ANOVA, F_1,31_ = 0.9, *P = *0.349; Table S1) or sterile soils (ANOVA, F_1,31_ = 0.006, *P = *0.937; Table S1). The addition of aflatoxin had no effect on fitness in natural (ANOVA, F_1,31_ = 2.5, *P = *0.124; Table S1) or sterile soil (ANOVA, F_1,31_ = 0.66, *P = *0.8; Table S1), regardless of chemotype.

### (iii) Experiment 3.

**FIG 3:**
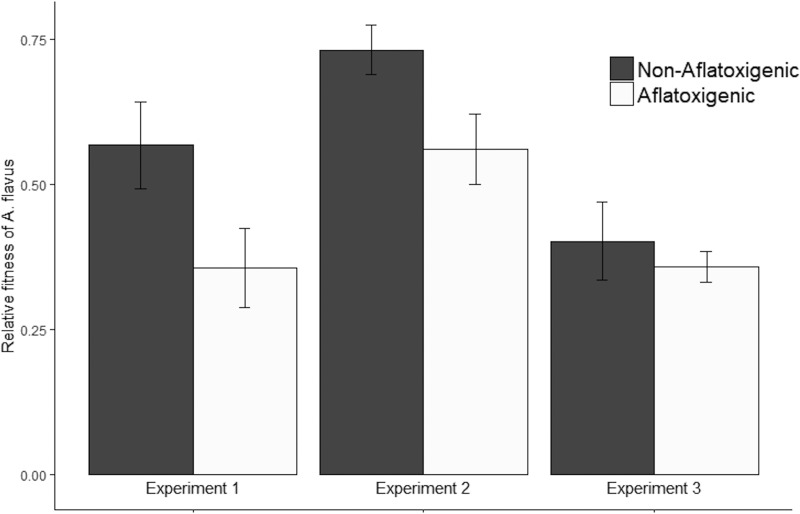
Mean relative fitness of aflatoxigenic and nonaflatoxigenic isolates of Aspergillus flavus from experiments 1, 2, and 3, all in natural soil at 37°C. Combined data from the three experiments included totals of 27 aflatoxigenic and 11 nonaflatoxigenic clone-corrected isolates. The fitness of *A. flavus* was estimated by qPCR relative to standard curves. Each microcosm was replicated twice and incubated for 4 days. Error bars represent the SE. The fitness of aflatoxigenic isolates was significantly lower than that of nonaflatoxigenic isolates across all experiments (*P = *0.05).

Since experiments 1 and 2 unexpectedly showed that aflatoxigenic isolates had lower fitness than did nonaflatoxigenic isolates in natural soil, we estimated the fitness, in natural soil, of an additional sample of 20 aflatoxigenic and seven nonaflatoxigenic A. flavus field isolates. In this experiment, the mean fitness of aflatoxigenic and nonaflatoxigenic isolates was not significantly different (ANOVA, F_1,25_ = 0.13, *P = *0.72; Table S1; Fig. 3). However, when A. flavus fitness data (from natural soil incubated at 37°C without aflatoxin added) from experiments 1 to 3 were combined, aflatoxigenic isolates had significantly lower fitness than nonaflatoxigenic isolates (ANOVA, F_1,39.9_ = 4.1, *P = *0.050; Table S1). In this combined analysis, there was a significant effect of the experiment blocking variable on fitness (ANOVA, F_2,82.7_ = 6.6, *P = *0.002; Table S1). This effect of experiment on fitness, however, did not interact with the effect of chemotype (ANOVA, F_2,82.7_ = 1.4, *P = *0.242; Table S1).

The population that isolates originated from (population A or B in reference 22) did not have a significant effect on fitness (ANOVA, F_1,76.8_ = 0.794, *P = *0.376; Table S1). We found no evidence of genetic differentiation between isolates of different chemotypes used in this study (analysis of molecular variance [AMOVA] Ф_PT_ = 0, *P = *0.534) nor any evidence that genetic relatedness was significantly greater within chemotypes than between chemotypes (Mantel test, *r =* −0.021, *P = *0.538). We thus find no evidence to support the hypothesis that other differences in the genetic background are causing a systematic difference in fitness, leaving us to conclude that aflatoxin production *per se* is the most parsimonious explanation for the observed differences.

### Effects of aflatoxin on soil microbial communities.

**FIG 4:**
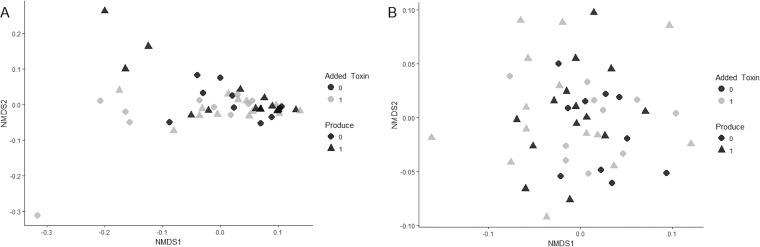
Nonmetric multidimensional scaling (NMDS) analysis representing similarity in fungal community (A) and bacterial community (B) composition in soil microcosms incubated with (black) and without (gray) the addition of aflatoxin after inoculation with aflatoxigenic (triangles) or nonaflatoxigenic (circles) isolates of *A. flavus*. Results are from amplicon sequencing of DNA samples from natural soils in experiment 2.

Amplicon sequencing of fungal internal transcribed spacer (ITS) and bacterial small subunit (SSU) rRNA genes from natural soils in experiment 2 revealed that the alpha diversity of the fungal community, as measured by the Shannon index, was unaffected by either the addition of aflatoxin to soil (mean ± standard deviation [SD], 1.6 ± 0.37 and 1.58 ± 0.49 with and without aflatoxin, respectively) or the chemotype of isolates (1.53 ± 0.48 and 1.67 ± 0.34 for aflatoxigenic and nonaflatoxigenic isolates, respectively) (ANOVA, F_1,44_ = 0.017, *P = *0.898, and F_1,44_ = 0.033, *P = *0.259, respectively; Table S2). Similarly, bacterial community alpha diversity was indistinguishable for aflatoxin amendments (5.98 ± 0.09 and 5.99 ± 0.11 with and without added aflatoxin, respectively) and chemotype (5.98 ± 0.09 and 5.99 ± 0.11 for aflatoxigenic and nonaflatoxigenic isolates, respectively) (ANOVA, F_1,44_ = 1.15, *P = *0.289 and, F_1,44_ = 2.55, *P = *0.118, respectively; Table S2). In addition, fungal beta diversity was unaffected by chemotype (permutational multivariate analysis of variance [PERMANOVA], *R*^2^ = 0.03, *P* = 0.144; Table S3), though there was a small but significant effect of chemotype on bacterial beta diversity (PERMANOVA, *R*^2^ = 0.03, *P = *0.048; Table S3 and Fig. 4). Analysis of all bacterial and fungal operational taxonomic units (OTUs), including all OTUs annotated as Bacillus spp., indicated no change in the relative abundances of individual taxa between treatments (Fig. S1). We thus conclude that neither aflatoxin production nor the addition of aflatoxin to soil had a significant effect on the fungal or bacterial communities in natural soil.

10.1128/mBio.02782-18.4 TABLE S2 Results of ANOVA model for Shannon index of fungal and bacterial communities as a function of added toxin and chemotype of *A. flavus*. Download Table S2, DOCX file, 0.01 MB. Copyright © 2019 Drott et al. 2019 Drott et al. This content is distributed under the terms of the Creative Commons Attribution 4.0 International license.

10.1128/mBio.02782-18.5 TABLE S3 Results of PERMANOVA model of fungal and bacterial community composition as a function of added toxin and chemotype of *A. flavus*. Download Table S3, DOCX file, 0.01 MB. Copyright © 2019 Drott et al. 2019 Drott et al. This content is distributed under the terms of the Creative Commons Attribution 4.0 International license.

10.1128/mBio.02782-18.6 FIG S1 Results of DESeq analysis (3) showing the change in abundance of individual fungal (A and C) and bacterial (B and D) OTUs between soil microcosms inoculated with aflatoxigenic and nonaflatoxigenic (A and B) *A. flavus* isolates and microcosms with and without added aflatoxin (C and D). None of the changes in OTU abundance are statistically significant. NA, fungal OTUs that could not be confidently assigned to a particular phylum. Download FIG S1, DOCX file, 0.9 MB. Copyright © 2019 Drott et al. 2019 Drott et al. This content is distributed under the terms of the Creative Commons Attribution 4.0 International license.

## DISCUSSION

Balancing selection for aflatoxin production is evident from the persistent polymorphism in the ability to produce aflatoxin among isolates of A. flavus found together in the same field (9, 22) and in molecular signatures in the aflatoxin gene cluster (10, 11). Recently, Drott et al. (13) provided experimental support for Janzen’s (12) hypothesis that aflatoxigenic isolates of A. flavus have a fitness advantage over nonaflatoxigenic isolates because of interference competition with some insects. Because soil is often reported as the natural habitat of A. flavus (9, 17–19, 25), we tested the hypothesis that aflatoxin production would also benefit aflatoxigenic isolates through interference competition with soil microbes. Contrary to expectations, however, aflatoxigenic isolates have lower fitness than nonaflatoxigenic isolates in the presence of microbes but not in sterile soil. Neither the addition of aflatoxin nor the aflatoxin-producing ability of the fungus (chemotype) had an effect on overall microbial community composition or on the relative abundance of specific taxa. If aflatoxin conferred a benefit because of interference competition with microbes, we would have expected to see changes in the composition of microbial communities. Therefore, we attribute the difference in fitness between chemotypes not to interference competition but to a metabolic cost of producing aflatoxin in natural soil. We speculate that this fitness cost can be detected in natural soil because of low-nutrient conditions caused by competition with microbes, whereas the relative cost of aflatoxin production may be smaller and not detectable when conditions are more favorable, as in sterile soil. Thus, in natural soil, in the presence of microbes, aflatoxigenic individuals may be selected against relative to nonaflatoxigenic individuals. Coupled with the findings of Drott et al. (13), our study suggests that competition with insects selects for aflatoxigenic isolates, whereas interaction with soil microbes selects for nonaflatoxigenic isolates. Together, these selective forces may maintain the polymorphism observed for aflatoxin production in A. flavus by balancing selection.

Little is known about the role of secondary metabolites in the ecology of soil microbes (30). The potential role of aflatoxin as an agent of interference competition in the soil ecology of A. flavus has previously been questioned because aflatoxin is quickly degraded in soil (31). However, in pure culture and in soil, large amounts of aflatoxin (media containing ≥10,000 ppb aflatoxin) inhibit the growth of some bacteria (26–28) and fungi (28). Although we did not quantify microbial populations overall, we did not find that the addition of pure aflatoxin or the production of aflatoxin by A. flavus had any effect on the soil microbial community composition. It is possible that our observation that aflatoxin had little impact compared to previous studies was due to our use of lower aflatoxin concentrations, which more closely resemble those found in soil. Given past observation of the expression of aflatoxin biosynthetic genes in soil (25), we assume that with the low A. flavus biomass we observed in soil, aflatoxigenic isolates produce aflatoxin at rates that do not typically keep up with the degradation of aflatoxin we observed, even under sterile conditions (Fig. S2). Such degradation may prevent the accumulation of measurable amounts of toxin in soils, perhaps minimizing a role for aflatoxin in interference competition with soil microbes. The shorter duration of our experiments (4 days) relative to previous experiments (70 days) may also explain in part why we did not see changes in microbial communities. A. flavus is relatively fast-growing and quickly colonizes organic matter that may fall to the soil surface while not growing into the soil itself (20). Given this life history, we believe that the shorter timeline of our study is representative of the ecology of A. flavus, although aflatoxin may impact soil microbial community structure over larger time scales not examined here.

10.1128/mBio.02782-18.7 FIG S2 Degradation of aflatoxin over time in sterile soils (triangles) and natural soils (circles) incubated at three temperatures. The amount of aflatoxin remaining was measured over 20 days. Each point is the average of 3 replicates (*n *=* *90 in total). Error bars represent the SE. Download FIG S2, DOCX file, 0.03 MB. Copyright © 2019 Drott et al. 2019 Drott et al. This content is distributed under the terms of the Creative Commons Attribution 4.0 International license.

While we did not observe any effect of aflatoxin on the composition of soil microbial communities, it is clear that these communities greatly affect the growth of A. flavus. The fitness of A. flavus was unchanged between 25°C and 37°C in sterile soils; however, in natural soils, fitness was an order of magnitude lower at the cooler temperature (Fig. 1). This finding is consistent with reports of suppressive soils on some plant-pathogenic fungi, where competition with other soil microbes decreases the pathogen’s fitness (reviewed in reference 32). As we observed, the suppressiveness of soil microbes can be mediated by abiotic factors like temperature (reviewed in reference 33). In fact, Henry (34) speculated that the higher incidence of “take-all” disease of wheat (caused by the fungus Gaeumannomyces graminis var. tritici) at more northern latitudes and during colder parts of the season may partially be explained by the temperature dependence of the suppressive effect, which is diminished in colder temperatures. Analogously, we speculate that lower population density of A. flavus at cooler latitudes (21, 22, 35) may partially be explained by greater suppressive effects of soil microbes, as observed here. These results, however, do not elucidate any particular interaction between soil microbial communities and aflatoxin production by A. flavus. As the microbial suppression we observed appears to be independent of aflatoxin production, we speculate that the most likely explanation for the fitness cost we observed is the energetic cost of aflatoxin production.

Isolating the fitness effects of a phenotype controlled by a single gene (or gene cluster, in the case of aflatoxin) is extremely challenging, in part, because of complex ecological interactions and the “noise” associated with measuring fitness under conditions representative of the field (36, 37). Even when fitness differences are large, it has been difficult to show significant differences in fungal fitness associated with a single gene (38, 39), emphasizing the importance of our findings to understanding the ecological role of secondary metabolite production in nature. The two most common approaches compare isogenic lines differing by a single gene (e.g., mutants or isogenic lines created by backcrossing) or compare effects among individuals differing in phenotype that are randomly sampled from natural populations, as we did in this study. Both of these approaches have their limitations (40). While the creation of isogenic lines by backcrossing in A. flavus would be prohibitively laborious (41), the use of molecular techniques to create mutants is routinely used for understanding the role of specific genes in molecular or biochemical mechanisms. Despite its simple appeal, however, the use of mutants is considerably less appropriate when investigating fitness. The transformation process alone, even without disrupting any genes, has the potential to decrease fitness in some fungi (42). Rigorous controls and replication (see, e.g., reference 43) are essential to avoid effects on fitness from experimental artifacts. However, avoiding confounding effects of gene knockouts, even with proper controls, may not be possible. Secondary mutations to nontarget genes occurred regularly in strains of yeast with experimentally disrupted genes (44). Newer methods of mutagenesis involving CRISPR-Cas9 appear to cause similar secondary mutations in mouse and human cell lines (45). Therefore, fitness can be affected by both the disruption of a target gene and nontarget effects (nontarget gene disruptions or compensatory mutations in nontarget genes), making it difficult, if not impossible, to ascribe fitness effects to any one gene. Such difficulties are emphasized by a lack of substantiation of relationships between secondary metabolism and fitness inferred from mutants in natural populations. Wilkinson et al. (46) demonstrated that laboratory mutants that are deficient in sterigmatocystin (the immediate precursor to aflatoxin in the biosynthetic pathway) have reduced sporulation *in vitro*; however, several studies of natural isolates found no relationship between toxin-producing ability and fungal fitness (47, 48). Part of this difference may be explained by different effects on fitness of experimental gene disruptions compared to naturally occurring mutations in the same gene conferring the same phenotype (49). Furthermore, few studies compare the fitness of independently replicated mutants with the same gene disrupted in different ways, limiting comparisons to a single mutation in a single genetic background.

The most important limitation of assessing the fitness associated with a single gene (or gene cluster) by comparing individuals from natural populations, as we did in this study, is the potential for other genes that are in linkage disequilibrium (LD) with the gene of interest to contribute to differences in fitness. Although there is evidence of ancient recombination (10, 11), given the clonal nature of A. flavus (9), we have to consider the contribution of LD to the differences we saw in fitness between aflatoxigenic and nonaflatoxigenic field isolates. Several lines of evidence suggest that LD may not be a major concern. For example, correlations between the production of aflatoxin and the production of other mycotoxins (50–52) and between aflatoxin production and fitness of A. flavus (47, 48, 53, 54) are contradictory between different studies. We speculate that the contradictory nature of studies examining correlations between aflatoxin production and other traits *in vitro* reflects that, if such correlations exist, they are likely to be weak when measured in a random sample of a natural population. In addition to a lack of evidence that there is any correlation between fungal fitness and aflatoxin-producing ability, in our study, neither the Mantel test nor AMOVA indicated any evidence of partitioning of genetic diversity based on chemotype (Fig. S3). This finding further corroborates our assertion that the fitness costs we observed (Fig. 3) are related to aflatoxin production *per se* rather than to other genes in the genetic backgrounds of clones. We cannot, however, fully rule out the possible confounding effect of LD, just as mutants with disrupted genes cannot be ensured of being free of secondary mutations or representative of naturally occurring mutations. The isolates we used are a random sample of A. flavus populations in the United States (Fig. S3) and thus are representative of populations in which balancing selection for aflatoxin production has been observed. Thus, if there is any LD among isolates used in this study, it is reflective of genetic associations in natural fungal populations.

10.1128/mBio.02782-18.8 FIG S3 Minimum-spanning network (MSN) representing the genetic relatedness of *Aspergillus flavus* isolates sampled by Drott et al. (22). Isolates used in this study were compared to all isolates sampled by Drott et al. (22) (A) and to each other based on aflatoxin-producing ability (B). Genetic distances were estimated from 10 microsatellite markers using Bruvo’s distance. Download FIG S3, DOCX file, 1.1 MB. Copyright © 2019 Drott et al. 2019 Drott et al. This content is distributed under the terms of the Creative Commons Attribution 4.0 International license.

10.1128/mBio.02782-18.9 DATA SET S1 All resulting data from experiments conducted in this study. Download DATA SET S1, XLSX file, 1.7 MB. Copyright © 2019 Drott et al. 2019 Drott et al. This content is distributed under the terms of the Creative Commons Attribution 4.0 International license.

To our knowledge, these results are the first evidence of a fitness cost of the production of aflatoxin in A. flavus. This finding also represents a rare piece of evidence for the cost of secondary metabolites produced by microbes in general. When coupled with previous findings that aflatoxin production benefits A. flavus in the presence of some insects (13), this opposing selective force may explain the maintenance of chemotype polymorphisms (8) and signatures of balancing selection observed in the aflatoxin gene cluster (10). We speculate that when A. flavus competes with soil microbes for small pieces of organic matter (as used in this study), and in the absence of insects or other invertebrates, aflatoxin does not provide any fitness benefit and is costly to produce. This cost, however, may be negligible (or too small to detect) when nutrients are plentiful, as on laboratory media *in vitro* or in microcosms with sterile soil. Conversely, the cost of aflatoxin production may be outweighed by a fitness advantage when insects are present (13). Although these studies together create a theoretical framework that explains balancing selection, they do not preclude other fitness benefits or costs of aflatoxin production that have yet to be measured. Furthermore, it is unclear what impact, if any, the forces demonstrated in laboratory microcosms have under natural conditions. Our finding of a fitness cost associated with aflatoxin production where we hypothesized a fitness advantage emphasizes the need for understanding the biology of organisms in an ecological context rather than relying only on inferences from molecular genetic analysis of genome sequences or the performance of mutants *in vitro*. Indeed, while there have been several papers presenting hypotheses and inferences about patterns observed in fungal secondary metabolites from genome sequences (55–57), there is a dearth of information on the relevant biology and ecology of the organisms in which they are observed. Our study is one step in filling such gaps.

### Data accessibility.

The data sets supporting this article have been uploaded as part of the supplemental material.

## MATERIALS AND METHODS

### Cultures of Aspergillus flavus.

To study the effects of aflatoxin production on the fitness of A. flavus, we used a random sample of aflatoxigenic and nonaflatoxigenic field isolates that was stratified by state to maximize geographic distribution. Aspergillus flavus was isolated between 2013 and 2017 from independent soil samples from corn fields in Pennsylvania, North Carolina, Florida, Texas, and Oklahoma (Table S4) using dilution-plating methods described by Drott et al. (22). All isolates were genotyped with microsatellite markers to determine their genetic relatedness (22) after some of the experiments described below were completed.

10.1128/mBio.02782-18.1 TABLE S4 Mycotoxin production, multilocus genotype, and origin of isolates used in experiments. Download Table S4, DOCX file, 0.02 MB. Copyright © 2019 Drott et al. 2019 Drott et al. This content is distributed under the terms of the Creative Commons Attribution 4.0 International license.

Isolates were cultured in yeast extract-sucrose (YES) medium (8) and on Drosophila culture medium (DCM) (13) to determine aflatoxin chemotype by high-performance liquid chromatography (HPLC), as described previously (13). Cultures on DCM were mechanically damaged with a sterile toothpick to stimulate greater production of aflatoxin (13). Both of these assays were replicated twice for all isolates.

### Experimental soil microcosms.

Soil used in microcosms was collected from the top 2 cm of an agricultural field in Ithaca, NY. Soils from this field are characterized as Langford channery silt loam, with 2 to 8% slopes, eroded. Soil was air-dried at room temperature for 4 days, passed through a 0.5-mm sieve to homogenize the sample, and stored at 4°C for no longer than 3 months before use. While several Aspergillus spp. were commonly observed in this field soil, repeated attempts to isolate and detect A. flavus by quantitative PCR (qPCR) (described below) indicated that A. flavus was not present. Autoclaved soil was used in experiments where sterile soil was needed.

Soil microcosms consisted of 5 g soil in 50-ml plastic screw-cap tubes. To each tube we added 0.3 g corn meal that was sieved to obtain particles between 0.25 and 0.5 mm in size. We conducted blind studies in which fungal isolates were assigned a random number independent of relevant metadata until after analysis was completed. A. flavus inoculum was added to soil microcosms in 410 μl H_2_O at 120 spores/μl (for a final concentration of ∼10,000 spores/g soil; see Supplemental Methods in Text S1 in the supplemental material). Spore counts obtained using a hemocytometer were validated by plating a small volume of diluted suspension on potato dextrose agar and counting the number of resulting colonies. This spore density is consistent with results from several studies that have quantified A. flavus in field soils (58). Furthermore, A. flavus is often wind-dispersed (59), resulting in the dispersal of spores into already-established microbial communities; our microcosms simulate such dispersal.

10.1128/mBio.02782-18.10 TEXT S1 Supplemental methods and references. Download Text S1, DOCX file, 0.02 MB. Copyright © 2019 Drott et al. 2019 Drott et al. This content is distributed under the terms of the Creative Commons Attribution 4.0 International license.

### Quantitative PCR for estimating fitness of A. flavus in soil microcosms.

After 4 days of incubation, soil microcosms were homogenized by vortexing three times for 30 s each, with vigorous shaking by hand after each vortex. DNA was extracted from 0.25 g of soil using the Mo Bio PowerSoil kit (Mo Bio Laboratories, Solana Beach, CA), following the manufacturer’s DNeasy PowerSoil protocol for low biomass soil with RNase. We modified the protocol with the addition of three phenol-chloroform-isoamyl alcohol (24:8:1) extractions after the initial vortex, and the addition of a 600-μl 70% ethanol wash of the spin column immediately before the elution of DNA.

We quantified DNA of A. flavus by qPCR as a proxy for fungal fitness using primers, reaction conditions, and methods previously described (13). We used a genetic marker in the *omtA-1* gene in the aflatoxin biosynthetic cluster (13). Tests of several other PCR primer pairs were not specific enough to A. flavus to reliably quantify its biomass in field soil (Table S5). Use of *omtA-1* resulted in 11 nonaflatoxigenic isolates whose DNA could not be amplified with this marker, presumably because this gene has been deleted (60).

10.1128/mBio.02782-18.2 TABLE S5 qPCR primers that were tested for specificity for *Aspergillus flavus* DNA and control soil DNA containing no *A. flavus*. Download Table S5, DOCX file, 0.02 MB. Copyright © 2019 Drott et al. 2019 Drott et al. This content is distributed under the terms of the Creative Commons Attribution 4.0 International license.

Standard curves were constructed by pooling three randomly selected experimental DNAs and creating a dilution series, as described previously (13). A subset of DNAs were also chosen at random to confirm qPCR efficiency under all experimental conditions. All efficiencies were between 90 and 100% with *r*^2^ of >0.99. Standard curves were identical for all plates within an experiment but not between experiments.

Quantification of fungal DNA by qPCR as a proxy for fungal fitness was used previously to compare the fitness of aflatoxigenic and nonaflatoxigenic isolates (13). While A. flavus may produce sclerotia, conidia, and mycelia, to the best of our knowledge, there is no clear association between sclerotial and conidial phenotypes and aflatoxin production within the L-strain of A. flavus. Furthermore, observation of any sclerotia being formed in our microcosms in the 4-day period was rare, and DNA in all structures would likely contribute to our measure of fitness. As our method allows for quantification of total growth including allocation of resources to all potential fungal structures, it is consistent with the recommendations of Pringle and Taylor (61), who suggest that a single measure of fitness, such as ours, is sufficient for ecological questions about fungi with complex life histories.

### HPLC for quantifying aflatoxin from soil.

Aflatoxin was extracted from total soil remaining after DNA extraction by adding 2.5 ml sterile deionized H_2_O and 7.5 ml ethyl acetate to each microcosm tube (25). The mixture was shaken overnight at 100 rpm on an orbital shaker (Lab-Line, Melrose Park, IL) and centrifuged at 2,500 × *g* for 10 min, and 6 ml of the organic layer was reduced to dryness in a silanized tube under a nitrogen stream. Aflatoxin was dissolved in 1 ml of 45% methanol and quantified by HPLC as described previously (13), except for the use of a Zorbax Eclipse XDB C_18_, 4.6 by 150-mm, 3.5-μm column (Agilent Technologies, Santa Clara, CA, USA) at a flow rate of 1 ml/min to improve detection. Yields, as tested by adding pure aflatoxin diluted in methanol to soil, were ∼40%. Reported aflatoxin concentrations (in parts per billion [ppb]) have been corrected for 40% yield. Tests of aflatoxin degradation in natural and sterile soils were also conducted (Supplemental Methods in Text S1 and Fig. S1).

### Effects of aflatoxin on fitness of A. flavus in soils.

The effect of chemotype on A. flavus fitness was tested under various soil conditions in three experiments, explained below.

**(i) Experiment 1.** In experiment 1, to test the effect of the interaction of temperature and presence of soil microbes on the fitness of both chemotypes, four aflatoxigenic and three nonaflatoxigenic isolates were randomly assigned to separate microcosms with sterile or natural soils and incubated at 25, 37, and 42°C for 4 days. These temperatures are known to be suboptimal, optimal, and superoptimal, respectively, for both growth and production of aflatoxin by A. flavus (62). Furthermore, the lower two temperatures (25 and 37°C) are similar to average soil temperatures (34°C) observed in agricultural regions where A. flavus is common (19), while all temperatures used are reflective of air temperatures that the fungus may experience on the soil surface in these regions. Every isolate was replicated twice under each condition in a full factorial design. Additionally, a microcosm with no added A. flavus was included under each condition as a control. As we were most easily able to quantify differences between aflatoxigenic and nonaflatoxigenic isolates at 37°C, subsequent experiments were conducted at this temperature.

**(ii) Experiment 2.** To test the potential of added aflatoxin to affect the fitness of A. flavus, microcosms with sterile or natural soil were inoculated with spores from one of 12 A. flavus isolates in a second experiment. Each of seven aflatoxigenic and five nonaflatoxigenic isolates was replicated twice with and without 500 ng/g soil (ppb) aflatoxin added. The resulting microcosms were incubated at 37°C for 4 days. DNA samples from microcosms containing natural soil in this experiment were later used in amplicon sequencing analysis (see below).

**(iii) Experiment 3.** To further test patterns seen in experiments 1 and 2, we conducted a third experiment using only natural soils incubated at 37°C for 4 days. To ensure independence of isolates by avoiding the same clone, we used genotype data from 10 microsatellite markers developed by Grubisha and Cotty (63) from a study by Drott et al. (22). We randomly selected independent multilocus genotypes from this pool of isolates. Using these criteria, we compared seven nonaflatoxigenic and 20 aflatoxigenic genotypes, each replicated twice as described in experiment 1.

### Effects of aflatoxin on soil microbial communities.

To determine the effect of aflatoxin on soil microbial communities, we used DNA samples from the natural soils in experiment 2 described above. We constructed libraries of the V4 region of bacterial 16S rRNA and the internal transcribed spacer 1 (ITS1) region of fungal rRNA using methods similar to those of Kozich et al. (64) (Supplemental Methods in Text S1).

Libraries were sequenced at Cornell University’s Genomics Facility using the Illumina MiSeq v3 sequencing chemistry (2 × 300-bp reads) on an Illumina MiSeq instrument. The 16S rRNA gene and ITS1 amplicon reads were assembled with PEAR (minimum overlap, 50 bp; assembly probability, 0.001; Phred score cutoff, 30), and sequencing primers and adapters were removed using cutadapt version 1.14 and demultiplexed into individual samples with deML (65, 66). Operational taxonomic units (OTUs) were assigned using a 3% dissimilarity cutoff and identified using VSEARCH version 2.5.2 and PIPITS version 1.5.0 pipelines for 16S gene and ITS1 amplicons, respectively (67, 68). Taxonomic affiliations of 16S and ITS1 sequences were performed with the SINTAX algorithm within USEARCH version 9.2.64 (sintax cutoff 0.8) using the Greengenes version 13.8 or UNITE version 7.2 sequence database, respectively (69–72).

### Statistical analyses.

Differences in fitness were explained in a series of mixed linear models using main and interaction effects of chemotype, the random effect of isolate nested in chemotype, and where applicable, soil sterility, addition of aflatoxin, and temperature. All models to explain differences in fitness were analyzed using ANOVA (type III) with Satterthwaite approximation. When data from all natural soils incubated at 37°C without the addition of aflatoxin were combined, the random effect of clone-corrected genotype replaced the random effect of isolate, and a categorical grouping variable was added to indicate the experiment from which the data originated. In this analysis, we accounted for subdivision recently observed in the U.S. population of A. flavus (22) using the main effect of population as determined by Drott et al. (22).

Transformations using log10 and square root of response variables were necessary in some analyses (Tables S4 to S6) to equalize variances and linearize the relationship between response and predictor variables. Results were analyzed using R statistics 3.4.0 (73) packages ‘ARTool’ (74), ‘lmerTest’ (75), ‘lsmeans’ (76), ‘tidyverse’ (77), and ‘Rmisc’ (78) installed on 21 April 2017.

We also assessed the genetic relatedness of isolates used in this study based on microsatellite data from Drott et al. (22). To assess the potential for linkage disequilibrium (LD) between aflatoxin-producing ability and other genes as a confounding factor, we tested for genetic differentiation by chemotype using analysis of molecular variance (AMOVA). Additionally, we used the Mantel test to determine whether individuals were more closely related to other individuals in the same chemotype. Both of these tests were executed in GENALEX version 6.5 (79). Genetic relationships were mapped in minimum-spanning networks using *poppr* version 2.5.1 (80).

Microbial communities were analyzed for differences in alpha diversity (species diversity within samples) using an ANOVA (type III) of the Shannon diversity index as a function of the individual and interaction effects of aflatoxin production of the isolate and the addition of aflatoxin to the soil. Significant deviations in beta diversity (ratio of species diversity between groups) were tested using a PERMANOVA on a Bray-Curtis dissimilarity matrix using 1,000 permutations and identical model parameters as specified for alpha-diversity tests. Differential abundance of individual taxa between treatments was assessed using DESeq2 with a significance cutoff α of 0.05 with the default Bonferroni correction. This analysis was achieved using R packages ‘phyloseq’ (81), ‘ape’ (82), ‘ARTool’ (74), and ‘DESeq2’ (83).
