# Supplementary material for: Fitness Cost of Aflatoxin Production in Aspergillus flavus When Competing with Soil Microbes Could Maintain Balancing Selection
Source: mBio. 2019 Feb 19;10(1):e02782-18. doi: 10.1128/mBio.02782-18 (PMC6381279; doi:10.1128/mBio.02782-18)
Supplement: TABLE S1 [file mBio.02782-18-st001.docx]

|  |  | **Sum Sq** | **Mean Sq** | **NumDF** | **DenDF** | **F.value** | **Pr(>F)** |  |
| --- | --- | --- | --- | --- | --- | --- | --- | --- |
| **Experiment 1**^1^ | |  |  |  |  |  |  |  |
|  | Temp | 3.3827 | 1.6913 | 2 | 67 | 69.397 | <2.2e-16 | *** |
|  | Sterility | 4.706 | 4.706 | 1 | 67 | 193.092 | <2.2e-16 | *** |
|  | Chemotype | 0.0382 | 0.0382 | 1 | 5 | 1.568 | 0.26582 |  |
|  | Temp:Sterility | 0.6244 | 0.3122 | 2 | 67 | 12.809 | 1.95E-05 | *** |
|  | Temp:Chemotype | 0.0023 | 0.0011 | 2 | 67 | 0.046 | 0.95464 |  |
|  | Sterility:Chemotype | 0.1034 | 0.1034 | 1 | 67 | 4.242 | 0.04332 | * |
|  | Temp:Sterility:Chemotype | 0.0293 | 0.0147 | 2 | 67 | 0.602 | 0.55081 |  |
| **Experiment 2 Natural Soil** | | |  |  |  |  |  |  |
|  | Chemotype | 0.117568 | 0.11757 | 1 | 9 | 5.2013 | 0.4851 | * |
|  | PPB | 0.056511 | 0.05651 | 1 | 31 | 2.5001 | 0.12399 |  |
|  | Chemotype:PPB | 0.020403 | 0.0204 | 1 | 31 | 0.9026 | 0.34942 |  |
| **Experiment 2 Sterile Soil** | | |  |  |  |  |  |  |
|  | Chemotype | 0.081897 | 0.0819 | 1 | 9 | 1.37711 | 0.2707 |  |
|  | PPB | 0.003944 | 0.00394 | 1 | 31 | 0.06631 | 0.7985 |  |
|  | Chemotype:PPB | 0.000376 | 0.00376 | 1 | 31 | 0.00632 | 0.9371 |  |
| **Experiment 3**^2^ | |  |  |  |  |  |  |  |
|  | Chemotype | 0.006123 | 0.00612 | 1 | 25 | 0.13322 | 0.7182 |  |
| **Experiments 1, 2, 3**^2^ | | |  |  |  |  |  |  |
|  | Chemotype | 0.71609 | 0.71609 | 1 | 39.9 | 4.0897 | 0.04989 | * |
|  | Experiment | 2.31117 | 1.15559 | 2 | 82.7 | 6.5997 | 0.00219 | ** |
|  | Chemotype:Experiment | 0.50543 | 0.25271 | 2 | 82.7 | 1.4433 | 0.24204 |  |
| **Experiments 1, 2, 3 with population**^2^ | | | |  |  |  |  |  |
|  | Chemotype | 0.70513 | 0.70513 | 1 | 39.9 | 3.9531 | 0.05368 | . |
|  | Experiment | 2.21897 | 1.10948 | 2 | 81.67 | 6.22 | 0.00306 | ** |
|  | Population | 0.14159 | 0.14159 | 1 | 76.84 | 0.7938 | 0.37573 |  |
|  | Chemotype:Experiment | 0.50543 | 0.25271 | 2 | 82.7 | 1.4433 | 0.24204 |  |

^1^Biomass was transformed as sqrt(biomass) for this analysis
^2^ Biomass was transformed as log_10_(biomass) for this analysis
