# Supplementary material for: Fitness Cost of Aflatoxin Production in Aspergillus flavus When Competing with Soil Microbes Could Maintain Balancing Selection
Source: mBio. 2019 Feb 19;10(1):e02782-18. doi: 10.1128/mBio.02782-18 (PMC6381279; doi:10.1128/mBio.02782-18)
Supplement: TABLE S2 [file mBio.02782-18-st002.docx]

|  |  | **DF** | **Sum Sq** | **Mean Sq** | **F value** | **Pr(>F)** |
| --- | --- | --- | --- | --- | --- | --- |
| **Fungal** | |  |  |  |  |  |
|  | Added_Toxin | 1 | 0.003 | 0.00325 | 0.017 | 0.898 |
|  | Chemotype | 1 | 0.253 | 0.25308 | 1.306 | 0.259 |
|  | Added_toxinA:Chemotype | 1 | 0.271 | 0.27112 | 1.399 | 0.243 |
|  | Residuals | 44 | 8.528 | 0.19382 |  |  |
| **Bacterial** | |  |  |  |  |  |
|  | Added_Toxin | 1 | 0.013 | 0.012958 | 1.152 | 0.289 |
|  | Chemotype | 1 | 0.0287 | 0.028658 | 2.548 | 0.118 |
|  | Added_toxin:Chemotype | 1 | 0 | 0.000009 | 0.001 | 0.977 |
|  | Residuals | 44 | 0.495 | 0.011249 |  |  |
