# Supplementary material for: Fitness Cost of Aflatoxin Production in Aspergillus flavus When Competing with Soil Microbes Could Maintain Balancing Selection
Source: mBio. 2019 Feb 19;10(1):e02782-18. doi: 10.1128/mBio.02782-18 (PMC6381279; doi:10.1128/mBio.02782-18)
Supplement: TABLE S3 [file mBio.02782-18-st003.docx]

|  |  | **DF** | **Sum Sq** | **Mean Sq** | **F. Model** | **R2** | **Pr(>F)** |  |
| --- | --- | --- | --- | --- | --- | --- | --- | --- |
| **Fungal** | |  |  |  |  |  |  |  |
|  | Added_Toxin | 1 | 0.0487 | 0.048704 | 1.7729 | 0.03604 | 0.127 |  |
|  | Chemotype | 1 | 0.0448 | 0.044378 | 1.6155 | 0.03283 | 0.144 |  |
|  | Chemotype:Produce | 1 | 0.04976 | 0.049759 | 1.8113 | 0.03682 | 0.12 |  |
|  | Residuals | 44 | 1.20872 | 0.027471 |  | 0.89431 |  |  |
|  | Total | 47 | 1.35156 |  |  | 1 |  |  |
| **Bacterial** | |  |  |  |  |  |  |  |
|  | Added_Toxin | 1 | 0.0247 | 0.024705 | 1.07293 | 0.02272 | 0.271 |  |
|  | Chemotype | 1 | 0.03086 | 0.030863 | 1.34039 | 0.02838 | 0.048 | * |
|  | Chemotype:Produce | 1 | 0.01881 | 0.018805 | 0.81672 | 0.01729 | 0.942 |  |
|  | Residuals | 44 | 1.01313 | 0.02302 |  | 0.93161 |  |  |
|  | Total | 47 | 1.0875 |  |  | 1 |  |  |
