# Supplementary figures and images for: Fitness Cost of Aflatoxin Production in Aspergillus flavus When Competing with Soil Microbes Could Maintain Balancing Selection
Source: mBio. 2019 Feb 19;10(1):e02782-18. doi: 10.1128/mBio.02782-18 (PMC6381279; doi:10.1128/mBio.02782-18)

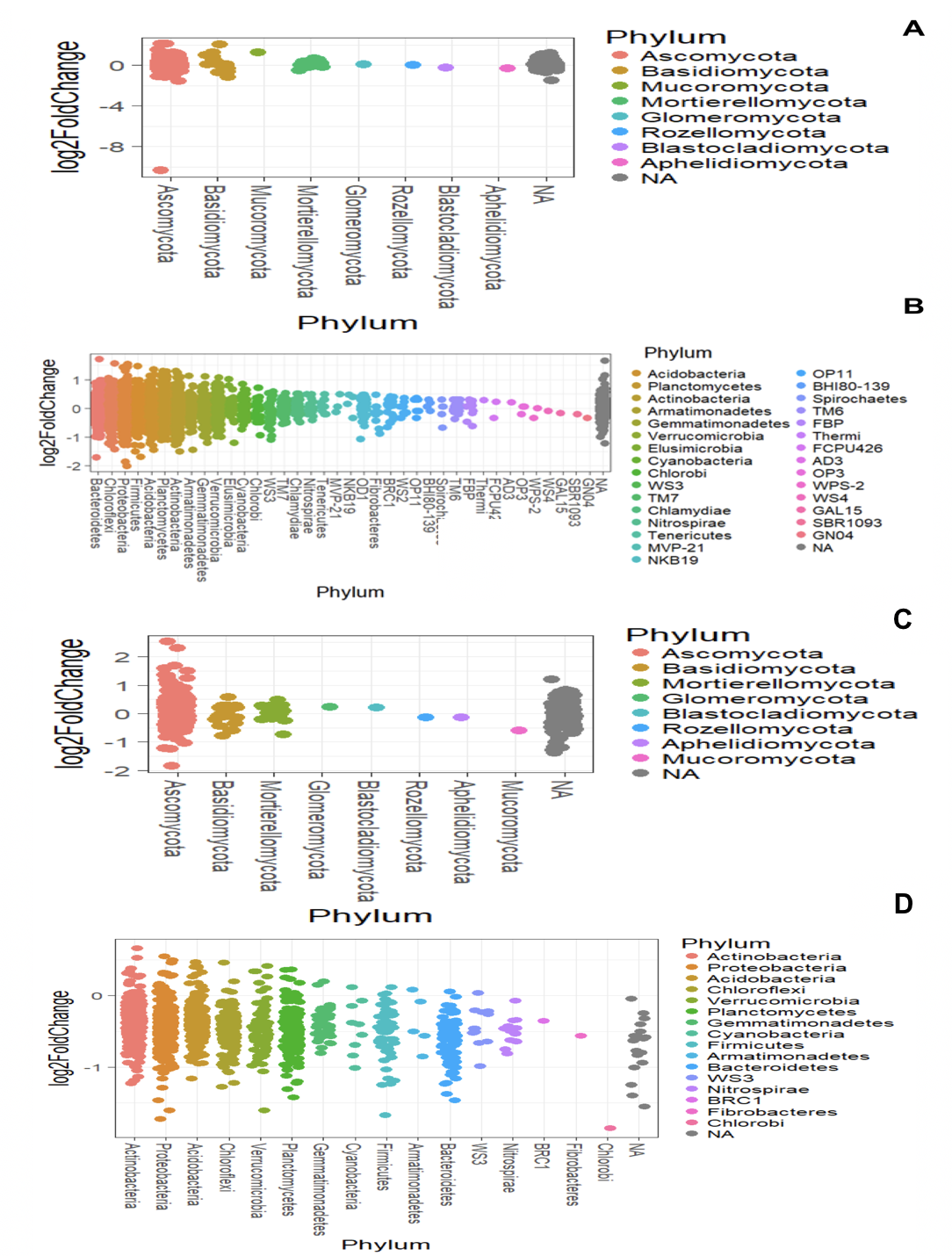

Supplement: FIG S1 [file mBio.02782-18-sf001.docx]

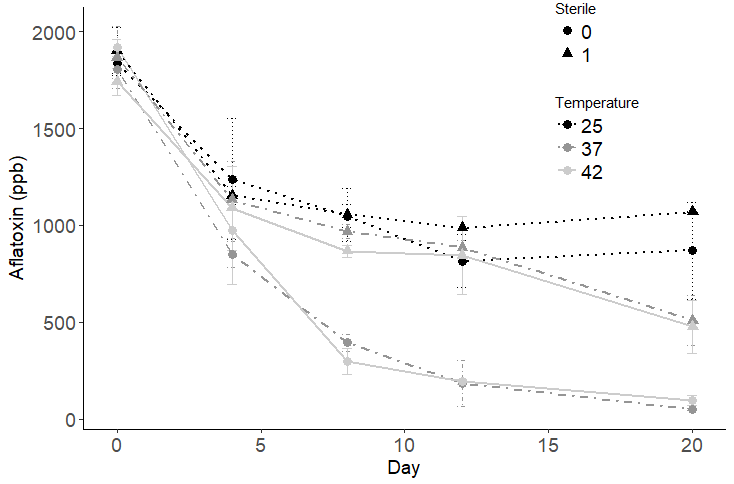

Supplement: FIG S2 [file mBio.02782-18-sf002.docx]

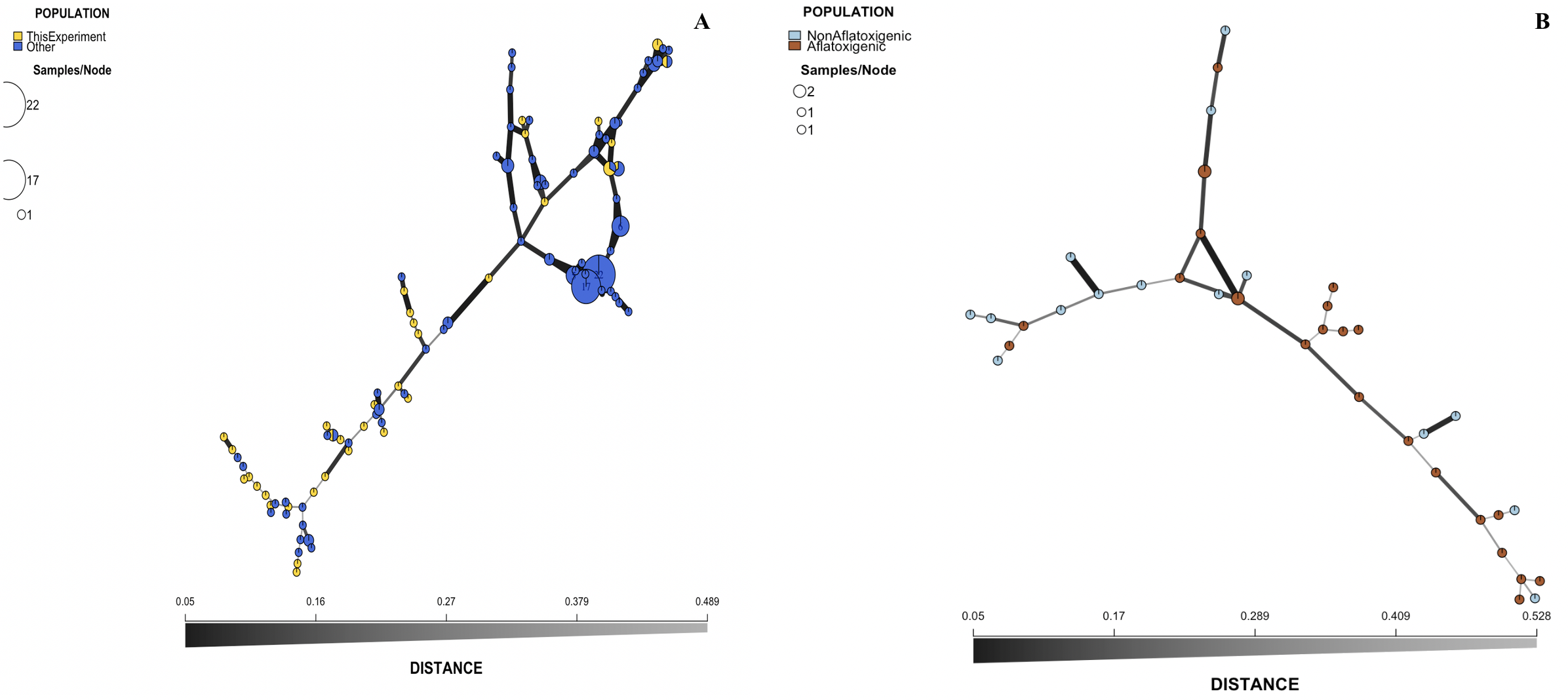

Supplement: FIG S3 [file mBio.02782-18-sf003.docx]
