# Supplementary material for: Fitness Cost of Aflatoxin Production in Aspergillus flavus When Competing with Soil Microbes Could Maintain Balancing Selection
Source: mBio. 2019 Feb 19;10(1):e02782-18. doi: 10.1128/mBio.02782-18 (PMC6381279; doi:10.1128/mBio.02782-18)
Supplement: TABLE S4 [file mBio.02782-18-st004.docx]

| Isolate | Aflatoxin | Origin^1^ | Town | Experiments^2^ | Genotype^3^ | Population^4^ |
| --- | --- | --- | --- | --- | --- | --- |
| B-25-1 | - | FL | Monticello | 1 | 22 | A |
| C-13-1 | + | FL | Quincy | 1 | 80 | A |
| C-19-3 | + | FL | Quincy | 1 | **48** | B |
| B-1-2 | - | PA | Manheim | 1 | **27** | A |
| B-16-2 | + | PA | Manheim | 1 | **23** | A |
| B-20-3 | + | PA | Manheim | 1 | 30 | B |
| A-15-3 | + | FL | Monticello | 2 | 42 | B |
| C-24-1 | - | FL | Quincy | 2 | 33 | B |
| D-2-8-2 | + | NC | Lexington | 2 | **37** | B |
| E-6-3 | + | NC | Goldsboro | 2 | 13 | A |
| A-22-2 | + | PA | Manheim | 2 | 81 | B |
| B-13-3 | - | PA | Manheim | 2 | **27** | A |
| B-16-3 | - | PA | Manheim | 2 | 24 | A |
| C-1-1 | + | PA | Manheim | 2 | 41 | B |
| B-7-1^5^ | - | FL | Monticello | 2 | 43 | B |
| A-11-1 | - | FL | Monticello | 3 | 31 | B |
| A-12-3 | + | FL | Monticello | 3 | 12 | A |
| A-22-2 | - | FL | Monticello | 3 | 5 | A |
| D-4-4-1 | + | NC | Lexington | 3 | 9 | A |
| E-14-1 | + | NC | Goldsboro | 3 | 20 | A |
| E-17-3 | + | NC | Goldsboro | 3 | **37** | B |
| E-25-3 | + | NC | Goldsboro | 3 | **48** | B |
| E-5-3 | - | NC | Goldsboro | 3 | 55 | B |
| E-6-1 | - | NC | Goldsboro | 3 | 10 | A |
| B-3-1 | + | OK | Tipton | 3 | 4 | A |
| B-4-1 | + | OK | Tipton | 3 | 8 | A |
| B-5-1 | + | OK | Tipton | 3 | 17 | A |
| B-6-1 | + | OK | Tipton | 3 | 15 | A |
| B-7-1 | + | OK | Tipton | 3 | 16 | A |
| B-1-1 | + | PA | Manheim | 3 | 26 | B |
| B-11-1 | + | PA | Manheim | 3 | **23** | A |
| B-7-1 | - | PA | Manheim | 3 | 85 | A |
| A-13-1 | + | TX | College Station | 3 | 53 | B |
| A-15-1 | + | TX | College Station | 3 | 51 | B |
| A-2-1 | + | TX | College Station | 3 | 2 | A |
| B-2-1 | + | TX | Caldwell | 3 | 3 | A |
| C-19-1 | + | TX | College Station | 3 | 29 | A |
| B-2-1 | + | FL | Monticello | 3 | 40 | B |
| B-14-2 | - | PA | Manheim | 1,3 | 25 | A |
| A-3-5-1 | - | NC | Lexington | 2,3 | 19 | A |
| B-11-1 | + | FL | Monticello | 2,3 | 18 | A |
| E-7-1 | + | NC | Goldsboro | 2,3 | 14 | A |

^1^PA, Pennsylvania; FL, Florida; NC, North Carolina; OK, Oklahoma; TX, Texas.
^2^ Refers to experiments in main text that isolates were used in.
^3^Genotypes that occur in more than one isolate are bolded.
^4^Refers to population as determined by Drott et al. (2018)
^5^Marked isolate did not amplify for qPCR, presumably because this non-aflatoxigenic isolate is missing the *omtA-1* gene that was targeted. The lack of amplification, however, was not determined until after the experiment had been run. This isolate was used in the amplicon sequencing experiment but was not included in estimates of fitness.
