# Supplementary material for: Fitness Cost of Aflatoxin Production in Aspergillus flavus When Competing with Soil Microbes Could Maintain Balancing Selection
Source: mBio. 2019 Feb 19;10(1):e02782-18. doi: 10.1128/mBio.02782-18 (PMC6381279; doi:10.1128/mBio.02782-18)
Supplement: TEXT S1 [file mBio.02782-18-s0001.docx]

Supplemental methods:

1. *Aspergillus flavus* inoculum preparation for soil microcosms.

Cultures of *A. flavus* were revived from lyophilized mycelium and grown on CZA at 30 °C in the dark for 5 d. Spores were harvested by flooding plates with 10 ml of sterile diH_2_O. Resulting spore suspensions were counted on a hemocytometer. Fungal inoculum from a single isolate was added to soil/corn meal mixture in 410 μl H_2_O at 120 spores/μl (for a final concentration of ~10,000 spores/g soil). All spore counts, and the viability of spores, was confirmed by plating an estimated 100 spores onto potato dextrose agar (PDA), incubating at 30 °C overnight, and counting the resulting number of colonies. In some experiments, high concentrations of aflatoxin were added to spore suspensions in 12 μl methanol per ml of spore suspension; non-aflatoxin controls received only methanol. Clumps of soil resulting from the addition of spore suspensions were homogenized manually using a sterile wooden applicator rod. Soils were distributed evenly along the length of the tube by gently shaking. Caps were loosened to allow for gas exchange and tubes were randomized into locations of a rack contained in a plastic bin, the bottom of which was flooded with water to decrease drying of microcosms.

1. Aflatoxin degradation in soil microcosms.

Soil microcosms were amended with aflatoxin as described above to a final concentration of 2000 ppb (ng aflatoxin/g dry soil). Both sterile and natural soils were replicated three times in a factorial design at 25, 37, and 42 °C and were incubated for 0, 4, 8, 12, and 20 d. Additionally a soil microcosm amended with methanol containing no aflatoxin was included at each condition as a control. Samples were stored at -20 °C until aflatoxin could be extracted from all tubes at the conclusion of the experiment. Differences in aflatoxin degradation were explained using a linear model with individual and interaction effects of the categorical variables: time, temperature, and soil sterility.

1. Library construction and amplicon sequencing.

We quantified DNA using PicoGreen dsDNA reagent (Thermo Fisher Scientific, Waltham, MA, USA) per the manufacturer’s instructions, and normalized to 10 ng/μL prior to PCR amplification. The V4 region of bacterial 16S ribosomal RNA (16S) genes and internal transcribed spacer 1 (ITS1) regions of fungal ribosomal RNA were amplified using primers 5’-GTGCCAGCMGCCGCGGTAA-3’ and 5’-GGACTACHVGGGTWTCTAAT-3’ and 5’-RCCWGYGGRRGGATCA-3’ and 5’-CTGCGTTCTTCATCGAT-3 (Martin and Rygiewicz 2005, Bokulich et al. 2013, Kozich et al. 2013), respectively. All PCR primers also included Illumina sequencing adapters and barcodes described in Kozich et al. (Kozich et al. 2013). Each PCR reaction was performed in triplicate 25-μl reactions consisting of 12.5 μl Q5 Hot Start High Fidelity Master Mix, 1.25 μl of each forward and reverse PCR primer (0.5 μM final concentration), 1.25 μl molecular grade bovine serum albumin (1 μg/μl final concentration), 1 μl DNA template, 0.625 μl 4X Quant-iT PicoGreen dsDNA reagent, and 7.125 μl of DNase-RNase free water. All PCR reactions were amplified on a CFX Connect Real-Time PCR detection system (Bio-Rad Laboratories, Hercules, CA, USA). Thermocycler conditions for 16S gene amplicons were one cycle of 95 °C for 2 min, 30 cycles of 95 °C for 20 sec, 55 °C for 15 sec, 72 °C for 10 sec, followed by a melt curve analysis from 76 °C to 95 °C using 0.5 °C sec^-1^ increments, and ending with a 72 °C incubation for 5 min followed by a 4 °C incubation. Fungal ITS1 thermocycler conditions were one cycle of 98 °C for 30 sec, 30 cycles of 95 °C for 5 sec, 50 °C for 20 sec, 72 °C for 10 sec, followed by a melt curve analysis from 75 °C to 95 °C using 0.5 °C sec^-1^ increments, and ending with a 72 °C incubation for 2 min and 4 °C incubation thereafter.

Triplicate PCR reactions were pooled and normalized to ~2 ng total DNA using a SequalPrep Normalization Plate (Thermo Fisher Scientific). The normalized DNA was combined into a single library and quantified using PicoGreen dsDNA reagent per the manufacturer’s instructions.

**Supplemental References:**

1. Grubisha L, Cotty P. Twenty-four microsatellite markers for the aflatoxin-producing fungus *Aspergillus flavus*. Molecular Ecology Resources. 2009;9:264–7.

2. Schuelke M. An economic method for the fluorescent labeling of PCR fragments. Nature Biotechnology. 2000;18(2):233-4.

3. Anders MILaWHaS. Moderated estimation of fold change and dispersion for RNA-seq data with DESeq2. Genome Biology. 2014;15(12):550.
