# Supplementary material for: Fitness Cost of Aflatoxin Production in Aspergillus flavus When Competing with Soil Microbes Could Maintain Balancing Selection
Source: mBio. 2019 Feb 19;10(1):e02782-18. doi: 10.1128/mBio.02782-18 (PMC6381279; doi:10.1128/mBio.02782-18)
Supplement: TABLE S5 [file mBio.02782-18-st005.docx]

| Primer Name | Study of Origin | Target | BP | *Aspergillus flavus* DNA | Control soil DNA | Primer Sequence |
| --- | --- | --- | --- | --- | --- | --- |
| Aflo8 | (Drott et al. 2017) | *omtA-1* | 125 | + | - | **F:** 5′ AGTGACAGAGCGTCCGAATC **R:** 5′ GGCGGTGACGATGTTAGAGA |
| Fla1/2 | (González-Salgado et al. 2008) | 18s rRNA | ~500 | + | + | **F:** 5’ GTAGGGTTCCTAGCGAGCC **R:** 5’ GGAAAAAGATTGATTTGCGTTC |
| AspIS/ITS4 | (Cruz and Buttner 2008) | ITS4 | ~230 | + | + | **F:** 5′ ATGCCTGTCCGAGCGT **R**: 5′ TCCTCCGCTTATTGATATGC |
| laeA | (Accinelli et al. 2009) | *LaeA* | 130 | + | + | **F:** 5’ GCTGGTACAATTTGGCTGTC **R:** 5’ CGCCTCCGACTTGACTTCTG |
| Flaq | (Sardiñas et al. 2011) | ITS2 | 100 | + | + | **F:** 5′ GTCGTCCCCTCTCCGG **R:** 5′ CTGGAAAAAGATTGATTTGCG |
| Af2 | (Mideros et al. 2009) | ITS1 | 73 | + | + | **F:** 5′ ATCATTACCGAGTGTAGGGTTCCT **R:** 5′ GCCGAAGCAACTAAGGTACAGTAAA |
| Act1 | (Drott et al. 2017) | *Act1* | 94 | + | + | **F**: 5′ GACGGAAGCCCCTATCAACC **R:** 5’ GATGGAGACGTAGAAGGCGG |
| omtB | (Accinelli et al. 2009) | *omtB* | 130 | - | + | **F:** 5’ AAGCAGATCATCCCAGTGAT **R:** 5’ CGAGTTGTGCCAGTTCAAAA |
